# Editorial: Advances and novel intervention in photodermatology

**DOI:** 10.3389/fmed.2025.1601630

**Published:** 2025-04-17

**Authors:** Yu Sun, Yunfan Du, Yiming Hu, Lintong Li, Yue Zheng

**Affiliations:** ^1^Department of Dermatology, Nanfang Hospital, Southern Medical University, Guangzhou, Guangdong, China; ^2^Department of Dermatology, The Third Affiliated Hospital of Sun Yat-sen University, Guangzhou, China

**Keywords:** skin photodamage, cellular landscape, non-coding RNAs, melanocytes, lasers and corticosteroids

The skin, as the largest organ in the human body, serves as the primary barrier against environmental stressors, which include ultraviolet (UV) radiation. Chronic exposure to UV radiation is a well-established cause of skin photoaging, characterized by wrinkles, pigmentation changes, loss of elasticity, and an increased risk of skin cancer. Recent research has significantly advanced our understanding of the molecular mechanisms underlying UV-induced skin damage, offering new insights into potential therapeutic strategies. This editorial synthesizes findings from four recent studies that explored the intricate processes of skin photoaging, the role of inflammatory factors, a novel *in vitro* platform to investigate melanocytes, and the efficacy of combined therapeutic approaches.

## The cellular landscape of photoaged skin

One notable investigation, led by Zhou et al., applied single-cell RNA sequencing to compare sun-exposed and unexposed skin from the same individual, revealing significant changes in cell populations and gene expression profiles. By comparing forearm (sun-exposed) and buttock (unexposed) skin from the same individual, the study identified 25 cell clusters and 12 skin cell types, with notable increases in sebaceous gland cells, keratinocytes, and melanocytes in sun-exposed skin. Conversely, fibroblasts, endothelial cells, and immune cells were reduced. This study highlights the dynamic changes in skin cell subsets induced by UV radiation, providing a detailed map of the cellular alterations associated with photoaging.

The researchers also found that UV exposure activated pathways such as MAPK, TNF-alpha, and TGF-beta, which are associated with inflammation and apoptosis. Their study further corroborates the role of inflammation in photoaging by identifying the activation of the IL-17 signaling pathway in multiple cell types, such as T cells, fibroblasts, and hair follicle cells. This pathway is associated with chronic low-level inflammation, or “inflammaging,” which is linked to immunosenescence and the accumulation of senescent cells. Activation of the PD-1/PD-L1 pathway in sun-exposed skin also suggests an immunosuppressive microenvironment that may contribute to the persistence of photoaged cells and the increased risk of skin cancer. These findings provide a detailed cellular map of photoaged skin and highlight potential therapeutic targets.

## miRNA and lncRNA interactions in UV damage

In the area of non-coding RNA, Chen et al. investigated the regulatory roles of miR-4298 and lncKRTAP5-6-3 in chronic UVB-induced damage in HaCaT cells, a human keratinocyte cell line. The researchers aimed to understand how these non-coding RNAs interact to influence the expression of Cathepsin D (CTSD), a lysosomal protease involved in skin repair and aging. By exposing HaCaT cells to repeated UVB radiation and manipulating miR-4298 levels, the study revealed that miR-4298 and lncKRTAP5-6-3 play synergistic roles in regulating the ERK-MAPK signaling pathway, which in turn affects CTSD expression. UVB exposure significantly reduced miR-4298 and lncKRTAP5-6-3 levels, while increasing ERK phosphorylation and decreasing CTSD expression. Overexpression of miR-4298 reversed these effects, suggesting its protective role against UVB-induced damage.

The findings highlight the importance of the miR-4298-lncKRTAP5-6-3-CTSD regulatory network in mitigating UVB-induced skin damage. miR-4298 was shown to upregulate lncKRTAP5-6-3, which in turn enhanced CTSD expression by inhibiting the ERK-MAPK pathway. This mechanism not only reduces oxidative stress and inflammation but also promotes skin repair by degrading harmful glycation end products (AGEs). These results provide new insights into the molecular mechanisms of skin photoaging and suggest that miR-4298 and lncKRTAP5-6-3 may serve as potential therapeutic targets for the prevention of UV-induced skin damage and related dermatological conditions.

## The role of melanocytes in photoprotection

The study by De Los Santos Gomez et al. highlighted the critical role of melanocytes in protecting the skin from the damaging effects of ultraviolet (UV) radiation. By comparing pigmented and non-pigmented skin equivalents exposed to repeated UV radiation, the researchers demonstrated that melanocytes, through the production of melanin, significantly reduce UV-induced damage. Pigmented skin equivalents exhibited less epidermal thinning, lower rates of apoptosis, and reduced DNA damage compared to non-pigmented equivalents. These findings underscore the importance of melanin as a natural photoprotective agent that absorbs UV radiation and minimizes oxidative stress, thereby preserving the structural integrity of the skin.

In addition to their role in absorbing UV radiation, melanocytes were found to modulate the skin's inflammatory response. Pigmented skin equivalents showed lower levels of pro-inflammatory cytokines, such as GM-CSF and IL-6, and higher levels of the anti-inflammatory cytokine IL-10. This suggests that melanocytes not only protect against UV-induced damage but also help regulate the skin's immune response, reducing inflammation and promoting tissue repair. The ability of melanocytes to balance inflammatory signaling further emphasizes their importance in maintaining skin health and resilience to environmental stressors such as UV radiation. These findings provide valuable insights into the multifaceted role of melanocytes in photoprotection and offer potential avenues for the development of therapies to mitigate UV-induced skin damage.

## Therapeutic interventions: combining lasers and corticosteroids

Functional melanocyte loss is one of the primary mechanisms underlying vitiligo, a chronic skin condition characterized by depigmentation. The study by Zhang et al. investigated the effectiveness of combining CO_2_ fractional laser therapy with compound betamethasone injections for the treatment of vitiligo. The researchers compared three treatment groups: Group A (CO_2_ laser + betamethasone), Group B (CO_2_ laser alone), and Group C (betamethasone alone). The results demonstrate that the combined therapy in Group A achieved a significantly higher total efficacy rate (92.73%) compared to Group B (74.55%) and Group C (67.27%). Additionally, Group A showed improved repigmentation rates, reduced relapse rates, and better quality of life and psychological wellbeing among patients. The study also found that the combined approach effectively modulated inflammatory factors by reducing pro-inflammatory cytokines (IL-17 and IFN-γ) and increasing anti-inflammatory cytokines (IL-10), which are crucial in the management of vitiligo.

The success of the combined therapy is attributed to the complementary mechanisms of the two treatments. The CO_2_ fractional laser promotes melanocyte proliferation and migration, while the compound betamethasone reduces inflammation and immune-mediated destruction of melanocytes. This synergy not only enhances repigmentation but also minimizes the risk of relapse, making it a promising strategy for the long-term management of vitiligo. The study highlights the importance of addressing both the structural and inflammatory aspects of vitiligo, offering a more comprehensive approach to treatment. These findings suggest that combining physical and pharmacological therapies can significantly improve outcomes for vitiligo patients, providing a new direction for clinical practice.

## Future directions

The integration of these studies provides a comprehensive view of the mechanisms underlying UV-induced skin damage and offers multiple avenues for therapeutic intervention. The single-cell transcriptomics approach by Zhou et al. offered unprecedented insight into the cellular changes induced by UV exposure, identifying specific cell populations and pathways that could be targeted to prevent or reverse photoaging. The study by Chen et al. on miRNA and lncRNA interactions provided a novel perspective on the molecular regulation of UV damage, suggesting that targeting these non-coding RNAs may offer new therapeutic opportunities.

Similarly, the protective role of melanocytes, as demonstrated by De Los Santos Gomez et al., suggests that enhancing melanocyte function could be a key strategy to prevent photoaging. Finally, the modulation of inflammatory pathways, as explored by Zhou et al. and Zhang et al., highlights the potential of combination therapies in the treatment of skin conditions such as vitiligo.

## Conclusion

The collective findings of these studies significantly advance our understanding of skin photodamage and offer promising directions for future research and therapy. By elucidating the roles of single-cell transcriptomics, miRNA/lncRNA interactions, melanocytes, and inflammatory factors, these studies provide a multi-faceted approach to combat UV-induced skin damage. Future research should focus on translating these insights into clinical applications, potentially leading to more effective treatments for photoaging and related skin disorders. As we continue to unravel the complex interplay of cellular and molecular mechanisms in skin photodamage, the potential for innovative and targeted therapies is becoming increasingly attainable.

